# Role of MicroRNA in linking diabetic retinal neurodegeneration and vascular degeneration

**DOI:** 10.3389/fendo.2024.1412138

**Published:** 2024-07-04

**Authors:** Haiyan Zhao, Yichen Cai, Junhua Pan, Qiu Chen

**Affiliations:** Hospital of Chengdu University of Traditional Chinese Medicine, Chengdu, Sichuan, China

**Keywords:** diabetic retinal degeneration, microRNA, linkage role, diabetic retinal neurodegeneration, vascular degeneration, diabetes

## Abstract

Diabetic retinopathy is the major cause of blindness in diabetic patients, with limited treatment options that do not always restore optimal vision. Retinal nerve degeneration and vascular degeneration are two primary pathological processes of diabetic retinopathy. The retinal nervous system and vascular cells have a close coupling relationship. The connection between neurodegeneration and vascular degeneration is not yet fully understood. Recent studies have found that microRNA plays a role in regulating diabetic retinal neurovascular degeneration and can help delay the progression of the disease. This article will review how microRNA acts as a bridge connecting diabetic retinal neurodegeneration and vascular degeneration, focusing on the mechanisms of apoptosis, oxidative stress, inflammation, and endothelial factors. The aim is to identify valuable targets for new research and clinical treatment of diabetic retinopathy.

## 1 Introduction

Diabetic retinopathy (DR) is a common complication of diabetes that can lead to retinal vascular and nerve degeneration, ultimately resulting in blindness for many patients. The prevalence of diabetes is increasing globally, with some regions predicted to have a prevalence rate of up to 15.7% by 2045 (1). Diabetic retinopathy is the most common cause of new cases of blindness in adults aged 20-74 years in developed countries (2). The optimal frequency and duration of drug injections and the regimen’s duration are still largely unknown. Steroids, vascular endothelial growth factor (VEGF)therapy, and laser therapy are the available treatments for advanced lesions, but these therapies come with potential side effects, and the risk of blindness remains for patients (3). Neurodegeneration and vascular degeneration of the retina are accompanied by hyperglycemia, with mechanistic crosstalk in apoptosis, oxidative stress, inflammation, and endothelial factors, but the exact pathways are unclear. Therefore, it is important to explore the mechanisms linking retinal neurodegeneration and vascular degeneration due to hyperglycemic states, to intervene in significant targets, and to develop therapeutic strategies to stop the progression of DR in its early stages.

MicroRNAs are involved in crosstalk in diabetic retinal neuropathy and vasculopathy. This article illustrates that microRNA ameliorates diabetic retinal neuropathy and vasculopathy by affecting apoptosis, oxidative stress, inflammation, and endothelial factors, to provide valuable targets for new research and clinical treatment of diabetic retinopathy.

## 2 Diabetic retinal neurodegeneration and vascular degeneration basic overview

Major risk factors for diabetic retinopathy are disease duration, hyperglycemia, hypertension, and dyslipidemia. DR causes leakage and blockage of retinal blood vessels (4). Two prominent manifestations of diabetic retinopathy are retinal neurodegeneration and vascular degeneration. Fundus manifestations in diabetic patients can be visualized by color fundus photographs, and fluorescein angiography, Optical Coherence Tomography(OCT) imaging (5). Fundus vascular microaneurysms, neovascularization, and fundus hemorrhage are the characteristics of fundus blood vessels in DR and are essential markers of DR staging (6). Leakage of inflammatory factors and plasma proteins due to disruption of the retinal barrier can result in the formation of hard exudates in the fundus. As the disease progresses, vasoconstriction and capillary occlusion lead to capillary damage and retinal ischemia (7), which can be observed as cotton wool spots. Extensive ischemic, neovascularization is defined as proliferative DR. New blood vessels at this stage are prone to rupture, which may eventually lead to retinal hemorrhage and detachment. In the end stage of diabetic retinopathy, severe hypoxia leads to neovascularization, vitreous hemorrhage, and retinal detachment (3). Diabetic macular edema (DME) is the most common complication in patients with DR, with a prevalence of 6.8% (8). In retinal neurodegeneration, a thinning of the peripapillary nerve fiber layer of the retina is found in diabetic retinopathy, while the protective effects of nerve growth factor (NGF) on retinal ganglion cells are disrupted (9). microRNAs regulate diabetic retinal neurovascular degeneration and slow disease progression.

## 3 Diabetic retinopathy and miRNA

### 3.1 Introduction to miRNA

miRNAs are ∼22 nt RNAs that contribute to regulating the expression of most mRNAs. miRNAs are single-stranded RNA molecules of approximately 19-24 nucleotides (nt) that are typically excised from RNA hairpin precursors of 60 to 110 nt. miRNAs can modulate the expression of specific target mRNAs by disincentive mRNA translation or promoting mRNA decay. miRNAs contribute to biological developmental processes, the maintenance of homeostasis, and the pathogenesis of diseases (10). Aberrant miRNA expression is linked to various diseases, such as cancer and neurological disorders. Therapeutic miRNA inhibitors are currently under development. Type RG-125/AZD4076, an anti-miRNA agent targeting miR-103/107, completed the first section of phase I clinical trial in type 2 diabetic subjects with non-alcoholic fatty liver disease.MRG-201 is a synthetic RNA oligonucleotide that targets and activates miR-29, Clinical trials have been conducted to evaluate the safety and efficacy of MRG-201 in the treatment of fibrotic diseases (11). The miRNA generation process includes transcription, cleavage by the microprocessor, nuclear export, processing by Dicer, and loading into the RNA-induced silencing complex (RISC). Decades of research have established a typical miRNA biogenesis pathway, starting with RNA polymerase II (RNAPII) transcription of miRNA host genes to produce primary miRNA (pri-miRNA) transcripts. Pri-miRNAs contain a localized stem-loop structure that encodes a miRNA duplex in the stem arm. The stem-loop is cleaved by the ribonuclease III enzyme Drosha and its RNA-binding protein cofactor DiGeorge syndrome critical region 8 (DGCR8), collectively known as the microprocessor complex, resulting in a 60-80 nt stem-loop/hairpin intermediate called a precursor miRNA (pre-miRNA). Nuclear export of pre-miRNAs is facilitated by Exportin 5 and Ran-GTP. In the cytoplasm, the second RNase III enzyme, Dicer, cleaves pre-miRNA end loops to produce miRNA duplexes. Each miRNA duplex produces two mature miRNAs: one from the 5 ‘station and one from the 3’ strand (12). In addition to the well-known miRNA biogenesis pathways, there are also non-classical pathways like the Microprocessor-Independent Pathway and Dicer-Independent Pathways, which contribute to a large collection of miRNAs.Regulatory post-translational modifications (PTMs) are important for miRNA regulation. PTM can affect the stability, localization and function of proteins, thereby influencing a wide range of cellular processes, and plays an important role in diabetes and its pathological consequences (13). Abnormal phosphorylation of pancreatic islet proteins is critical for the initiation and development of T2D. db/db Hyperactivation of GSK3 in pancreatic islets leads to phosphorylation of PDX1 and proteasomal degradation, which in turn leads to impaired β-cell function (14).

### 3.2 circulating miRNAs in diabetic retinopathy

miRNAs may be potential biomarkers for DR. miRNAs released into the circulation by cells can exist for about ≥ 2 weeks with strong stability. Exosomes can efficiently deliver miRNAs into recipient cells to influence biological responses in the paracrine/endocrine communication system (15). miRNAs are typically packaged in extracellular vesicles to prevent endogenous ribonuclease activity in the circulation and are present in this stable form. A systematic evaluation of microRNA expression in human diabetic retinopathy reported a total of 93 differentially expressed miRNAs. miR-320a was up-regulated in serum samples, and miR-423-5p was up-regulated in vitreous samples. miR-27b was down-regulated in serum samples (16). Liraglutide increases serum microRNA-27b, -130a, and -210 levels in patients with type 2 diabetes (17). Most miRNAs are intracellular, and specific intracellular microRNAs are associated with DR-associated cellular changes. Some microRNAs are extracellular, called circulating microRNAs. Circulating miRNAs are differentially expressed in the serum and body fluids of diabetic patients with and without retinopathy (18). Differential expression of circulating miRNAs was detectable in urine, serum, and plasma of diabetic patients. Circulating miRNAs such as miR-126, miR-150, miR-155, and miR-200b were dysregulated in patients with DR and preclinical animal models of DR, suggesting that circulating miRNAs may predict the progression of retinopathy from mild to vision-threatening (19).

## 4 Mechanisms of diabetic retinal neurodegeneration and vascular degeneration

### 4.1 Oxidative stress and apoptosis

Apoptosis and oxidative stress are essential mechanisms in diabetic retinal neurodegeneration. Diabetes selectively destroys cells that cannot increase the rate of glucose transport in a hyperglycemic environment. This intracellular hyperglycemia causes complications (20). The ischemic or hyperosmolar environment created by the hyperglycemic state in diabetic patients can cause oxidative stress. Oxidative stress is an imbalance between pro-oxidant and antioxidant, leading to an overproduction of reactive oxygen species (ROS). The retina is a high-energy-demanding organ highly susceptible to free radicals and ROS (21). Auto-oxidation of glucose, increased superoxide production/reduced scavenging, and formation of late glycosylation end products in diabetic states can increase oxidative stress, inducing apoptosis or death, impeding mitochondrial function, and causing damage to nerves, blood vessels, and retinal tissue. Excessive accumulation of ROS can result in mitochondrial damage, apoptosis, inflammation, lipid peroxidation, and structural and functional changes in the retina (22). ROS excess leads to mitochondrial dysfunction and triggers apoptosis, contributing to neovascularization (23). Oxidative stress induces retinal cell loss (24). Upregulation of Nicotinamide Adenine Dinucleotide Phosphorooxidase 4 under hyperglycemia and hypoxia conditions generates ROS, which impairs mitochondrial function and promotes retinal vasculopathy in diabetes mellitus (25). Oxidative stress affects Müller cell function, leading to increased ROS and mitochondrial alterations and dysregulated calcium responses (26).

### 4.2 Inflammatory effects

The retinal inflammatory response is mediated by specialized inflammatory cells within the retina also known as glial cells. Retinal glial cells consist of astrocytes, Muller cells, and microglia. These cells release inflammatory mediators when stimulated by hyperglycemia, oxidative stress, cell damage, polyol accumulation, advanced glycation end products (AGEs), and activated protein kinase C (PKC). Diabetes can lead to Muller cell death directly or activation of Muller cells through neural stress and cell death. Activated Muller cells are unable to maintain water homeostasis and cell barrier function and disrupt vascular endothelial growth factor, inducing inflammation and vascular leakage (27). Complement activation, AGE/Receptor of Advanced Glycation Endproducts activation, and inflammatory responses are observed in retinal pigment epithelial (RPE) cells of diabetic monkeys (28). It seems that in diabetic RPE cells, there is an activation of C3a/C3aR which acts as a negative regulator of inflammation. This leads to inhibition of the adenosine monophosphate signaling pathway and uncontrolled complement activation, which triggers a vicious cycle of inflammation. Nuclear factor kappa-B (NF-κB) controls the production of most pro-inflammatory cytokines, and in the high-glucose ARPE-19 cell model, the expression of NF-κB and inflammation-associated targets inducible nitric oxide synthase and tumor necrosis factor-alpha were reduced in the high-glucose group (29).

### 4.3 Vascular endothelial lesions

The etiology of DR is highly complex and involves several factors, including pericyte loss, basement membrane thickening, increased vascular permeability, and microaneurysm formation. Pericytes and endothelial cells are the two most common retinal vascular cells that play a crucial role in maintaining microvascular stability and remodeling (30). Pericytes are particularly important in regulating the blood supply to retinal tissue and visual perception (31). Diabetic retinal RPE cells exhibit pericyte loss and abnormal retinal vascular permeability. Insulin signaling in pericytes leads to the promotion of angiopoietin-1 secretion and endothelial Tie2 signaling, which in turn leads to excessive vascular overgrowth and abnormalities in the venous plexus (32). Increased vascular permeability and thickening of ganglion cells and the inner plexiform layer are key factors in the formation of retinal aneurysms, which may be caused by Angiotensin II (33). It has been demonstrated that down-regulation of VEGF-A levels hinders retinal vascularization in mice.

## 5 The relationship between diabetic retinal neurodegeneration and vascular degeneration

The American Diabetes Association has defined DR as a tissue-specific neurovascular complication involving the progressive destruction of the interdependence between multiple cell types in the retina. The metabolic needs of retinal neurons are preserved through the vascular system to maintain normal neurological function (34). When the good coupling between the nervous system and the vascular cells is disrupted, on the one hand, neuronal apoptosis and neuroglial degeneration release toxic mediators leading to disorders of the vascular system, and on the other hand, pathologic neovascularization fails to provide adequate nutritional support to the retinal nervous system. This eventually leads to irreversible late DR (35). RPE cells play a crucial role in communication between retinal photoreceptors and choroidal capillaries. Early dysfunction of RPE cells is associated with hyperglycemia (36). The ganglion cell bodies are sensitive to retinal ischemia (37). Photoreceptors in retina neurons may produce oxidative stress, which can lead to the production of pro-inflammatory factors and ultimately cause vasoconstriction.

Diabetic microangiopathy is highly related to neuronal degeneration. Patients with diabetes (with or without DR) have a significantly lower thickness of the retinal nerve fiber layer than those without diabetes, especially when associated with poorer metabolic control. With the duration of DM, perifoveal thickness may decrease. Poor metabolic control can result in thinning of the surrounding RNFL (38). It has also been suggested that diabetes initially affects retinal neurons, leading to neurodegeneration followed by more pronounced vascular abnormalities (39). In diabetic patients without DR, their color perception (40), visual field, and retinal sensitivity (41) function changed. In summary, different researchers have different standards for vascular degeneration and neurodegeneration. The connection between neurodegeneration and the onset of microvascular disease is significant. The cellular and molecular mechanisms that connect retinal neurodegeneration and microvascular diseases are still unclear, and more research is needed to understand the complex intercellular dynamics of healthy progression to diabetic retinopathy (42).

## 6 Treatment of diabetic retinopathy

Current treatment options for diabetic retinopathy include intravitreal administration of anti-VEGF drugs, laser therapy, steroids (such as dexamethasone or fluocinolone implants), and vitrectomy. Intravitreal anti-VEGF therapy is the preferred treatment for diabetic macular edema affecting the macular center (43). Anti-VEGF therapy with drugs like ranibizumab, bevacizumab, and abciximab has been proven to reduce diabetic macular edema and improve vision in many studies (44). VEGF treatment requires continuous intravitreous injection, and photocoagulation has obvious efficiency advantages. The standard treatment for proliferative diabetic retinopathy is total scattering retinal photocoagulation, which can effectively reduce the development of moderate and severe vision loss. Laser therapy can reduce the risk of moderate vision loss, but cannot improve the original vision. Several studies have indeed demonstrated the effectiveness of pan-retinal laser coagulation in preventing further progression of diabetic retinopathy. However, a significant number of patients still experience complications and visual impairment. There is no cure for diabetic retinopathy (45). Research has indicated that levels of miRNA in plasma and vitreous can fluctuate in patients with diabetic retinal neurovascular disease. These changes can help to slow down the progression of diabetic neurovascular disease by influencing the inflammatory response, regulating apoptosis, and affecting endothelial growth factors. This suggests that exocrine may play a crucial role in connecting diabetic vascular and neuropathy and could be a potential target for treating diabetic retinopathy.

miRNA can be used as a marker for early detection of diabetic retinopathy. Screening for miRNA in diabetic patients without retinopathy. Early detection of retinopathy precursors and timely blood pressure, lipid, and intensive glycemic control may have potential benefits in terms of vision preservation and improved quality of life for patients with diabetic retinopathy. Most of the effects of microRNAs on diabetic retinopathy are based on animal models, but there is a lack of research on clinical applications. The use of miRNA inhibitors or mimics for treating diabetic retinopathy in humans has not been approved (11). Animal model experiments have revealed that various types of miRNAs can have a protective effect against diabetic retinopathy, even though direct evidence of miRNA therapy in humans for vision protection and patient quality of life is lacking. Metformin, as a primary drug for diabetes treatment, not only directly regulates miRNA expression in tissues and organs, but also indirectly influences the development of miRNA-mediated tissues and organs through interactions with the circulatory system, vesicles, and viscera. Metformin can regulate the expression of miR-146a and miR-155, miR-21 and miR-155, and reduce inflammation. Emglicliflozin significantly reduced miR-21 and miR-92, alleviating endothelial dysfunction.miR-26a mimics delayed the thinning of the neural retinal layer and loss of ganglion number (46). Exosomes derived from mesenchymal stem cells (MSC-Exos) and expressing miR-126 significantly decreased the expression of HMGB1 and the activity of the NLRP3 inflammasome in human retinal endothelial cells (HRECs) exposed to high glucose. Additionally, mesenchymal (MSC) exosomes that overexpress miR-126 were capable of reducing levels of High mobility group box 1, NOD-, LRR- and pyrin domain-containing protein 3 inflammasome, and NF-κB/P65 proteins, leading to improvements in retinal endothelial dysfunction and inflammation (47). In a diabetic mouse model, intravitreal injection of miR-146 prevented diabetes-induced NF-κB initiation, microvascular leakage, and abnormal retinal function (48). Systemic injection of microRNA-467 antagonist prevents hyperglycemia-induced angiogenesis and growth in mice (49). In addition, research animal studies have shown that miRNA improves the symptoms of diabetic complications.

Exosomal ncRNAs derived from adipose stem cells (ADSC) can effectively relieve diabetic nucleic acid. miR-215-5p inhibits high glucose(HG)-induced Zinc finger E-box binding homeobox 2 (ZEB2) accumulation by directly targeting the 3’-UTR of ZEB2, thereby attenuating the HG-induced podocyte Endothelial to mesenchymal transition process and a series of pathological changes, and ultimately alleviating diabetic nephropathy (50) Serum and circulating exosomal miR-24-3p levels are significantly elevated in diabetic patients. Inhibition of miR-24-3p suppresses Human Umbilical Vein Endothelial cell proliferation, migration, and angiogenesis, leading to reduced apoptosis and enhanced wound healing (51). Injecting miR-10b-5p mimics or Klf11 small interfering RNA into diet-induced diabetic mice can rescue the diabetic gastroparesis phenotype in improving glucose homeostasis and gastrointestinal motility (52). Exosomes miR125a mimics reduced blood glucose levels, serum creatinine, urinary albumin-creatinine ratio, 24-hour urinary protein, and renal weight in DN rats (53). Insulin tolerance test and intraperitoneal glucose tolerance test showed that miR-20a-5p lowered blood glucose levels and ameliorated inflammation-induced diabetic myocardial fibrosis (54).

## 7 MicroRNA intensification of diabetic retinal neurodegeneration and vascular degeneration

### 7.1 Apoptosis and oxidative stress by MicroRNAs

**Figure 1:**
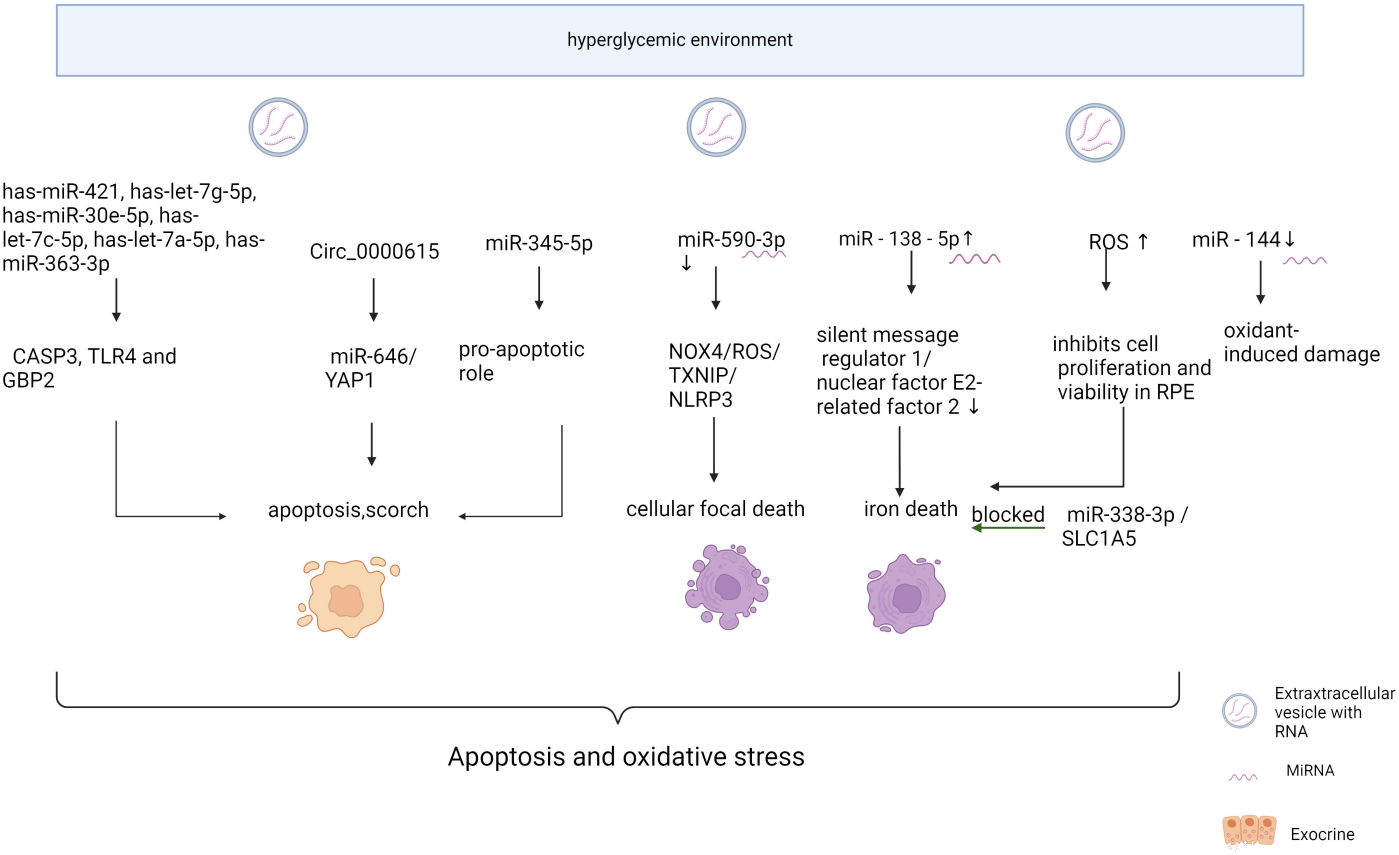
Mechanism of miRNA action in diabetic retinopathy through oxidative stress “Created with BioRender.com”.

In addition, there are many microRNA expression fluctuations due to changes in the hyperglycemic environment, which are involved in oxidative stress and apoptosis of the retina (**Figure 1**, **Table 1**). Methyl-CpG binding domain protein 2 (Mbd2) overexpression via miR-345-5p plays a pro-apoptotic role in high-glucose-induced retinal cell death (41). Oxidative stress is a critical factor in diabetic retinopathy. Studies have suggested that miRNAs such as has-miR-421, has-let-7g-5p, has-miR-30e-5p, has-let-7c-5p, has-let-7a-5p, has-miR-363-3p, has-miR-30c-5p, has-miR-98-5p, has-miR-224-5p, and has-miR-155-5p are involved in regulating three important inflammation-related genes: Caspase-3, Toll-like receptor 4, and Guanylate Binding Protein 2 (61) miR-590-3p downregulation promotes cellular focal death by the NOX4/ROS/TXNIP/NLRP3 pathway (55). Increased miR - 138 - 5p expression in RPE cells in high glucose environments leads to decreased activity of silent message regulator 1/nuclear factor E2-related factor 2, decreased expression of antioxidant response-related molecules, and increased iron death, which promotes cell death (56). Circ_0000615 affects high glucose-induced apoptosis, inflammation, and oxidative stress in human RPE cells via the miR-646/YAP1 axis (57). Up-regulated miR-338-3p targets Solute Carrier Family 1 Member 5, and reduces iron death and ROS production in RPE cells (45). Decreased expression of miR-144 in degenerative diseases of the brain and retina reduces the efficiency of antioxidant defense mechanisms (58). Overexpressing MiR-146a inhibited interleukin-6 (IL-6) signaling, leading to decreased Signal Transducer And Activator Of Transcription 3 (STAT3) and VEGF levels, resulting in reduced REC apoptosis (59). Metformin treatment increases the expression of miR-146a and miR-155 and reduces inflammation of endothelial cells (62). Troxerutin inhibits the inflammatory NF-κB pathway in the hippocampus of diabetic rats, possibly due to a negative feedback loop regulated by miR-146a. Troxerutin attenuates inflammatory effects and improves memory impairment in the hippocampus in diabetic complications (63). Resveratrol inhibits diabetes-induced down-regulation of miR-29b expression, rescues Müller cell apoptosis, and is a potential therapeutic option for DR (64). Upregulation of miR-200a-M inhibits Keap1/Nuclear factor erythroid 2-related factor 2(NRF2) signaling, which ultimately could attenuate hyperglycemia-induced inflammation and endothelial dysfunction (60). The above results suggest that microRNA affects retinal cell apoptosis and oxidative stress through a complex network of other cytokines.

**Table 1:** Mechanism of miRNA action in diabetic retinopathy through oxidative stress and apoptosis.

Name	Dysregulation	Possible signaling pathways	Pathogenic functions	Reference
miR-590-3p	downregulated	NOX4/ROS/TXNIP/NLRP3	promotes cellular focal death	(55)
miR-138- 5p	upregulated	silent message regulator 1/nuclear factor E2-related factor 2	Decreased expression of antioxidant response-related molecules and increased iron death	(56)
miR-646			apoptosis, inflammation, and oxidative stress in human RPE cells	(57)
miR-338-3p	upregulated	Solute Carrier Family 1 Member 5	reduces iron death and ROS production in RPE cells.	(45)
miR - 144	downregulated		reduces the efficiency of antioxidant defense mechanisms.	(58)
miR-146a	upregulated	inhibited IL-6 signaling	decreased STAT3 and VEGF levels, and reduced REC apoptosis.	(59)
miR-200a	upregulated	Keap1/NRF2 signaling	induced OS, inflammation, and endothelial dysfunction.	(60)

### 7.2 MicroRNAs regulate retinal inflammation through the NF-κB pathway

**Figure 2:**
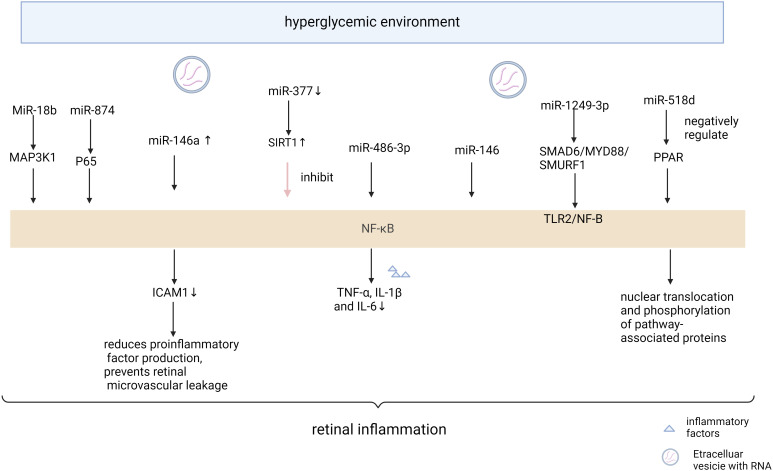
Mechanism of miRNA action in diabetic retinopathy through NF-κB pathway “Created with BioRender.com”.

The critical role of NF-κB signaling in the emergence of metabolic disorders has been well studied. NF-κB is an important regulator of many inflammatory chemokines and cytokines in a variety of metabolic tissues (65). The NF-κB signaling pathway is an important inflammatory pathway, and many MicroRNAs are involved in the signaling process of the NF-κB pathway (**Figure 2**, **Table 2**). miR-874 modulates the NF-κB signaling pathway by targeting p65 degradation to ameliorate retinopathy in diabetic rats (66). Increased expression of miR-146a leads to downregulation of the NF-κB downstream gene Intercellular cell adhesion molecule-1, which reduces the production of proinflammatory factors and prevents retinal microvessel leakage. miR-146’s negative feedback regulation of NF-kB activation may play a role in Tr-iBRB endothelial cells (48). Down-regulation of miR-377 may inhibit the NF-κB pathway and suppress the release of pro-inflammatory cytokines by directly up-regulating the expression of the target gene Silent information regulator 1 (SIRT1) (67). miR-486-3p affects the NF-kB pathway and decreases tumor necrosis factor-α, interleukin-1β, and interleukin-6 levels, thereby reducing diabetic retinal inflammation (68). MiR-18b inhibits NF-B p65 phosphorylation by targeting Mitogen-activated protein kinase kinase kinase 1. This reduces DR inflammation (69). Exosomal miR-1249-3p influences the development of SMAD6/MYD88/SMURF1 and consequently suppresses the Toll-like receptor 2/NF-B signaling pathway that mediates glucose homeostasis (70). The expression of Peroxisome proliferators-activated receptors is negatively regulated by miR-518d, which leads to the nuclear translocation process of NF-kB and phosphorylation of pathway-associated proteins, which leads to inflammatory responses (71). Therefore, the NF- κ B inflammatory signal pathway seems to be an important part of many miRNAs affecting retinal neuropathy and angiopathy. Expression of IL-1β, NF-κB, and VEGF were significantly increased in diabetic mice, and intravitreal delivery of miR-26a resulted in downregulation of the expression of these factors. Overexpression of miRNA-3976 led to increased apoptosis in Retinal Ganglion Cells-5 (RGC-5) cells and indirectly decreased the abundance of NFκB1 (72).

**Table 2:** Mechanism of miRNA action in diabetic retinopathy through NF-κB pathway.

Name	Dysregulation	Possible signaling pathways	Pathogenic functions	Reference
miR-874			promotes cellular focal death	(66)
miR-146a	upregulated	ICAM1	reduces the production of proinflammatory factors and prevents retinal microvessel leakage	(48)
miR-377	downregulated	SIRT1	suppress the release of pro-inflammatory cytokines	(67)
miR-486-3p	upregulated	TLR4/NF-κB	decreases TNF-α, IL-1β, IL-6levels	(68)
MiR-18b	upregulated	MAP3K1	reduces DR inflammation	(69)
miR-1249-3p	upregulated	SMAD6/MYD88/SMURF1	suppresses the TLR2/NF-B signaling	(70)
miR-518d	upregulated	NF-kB and phosphorylation of pathway-associated proteins	inflammatory responses	(71)

### 7.3 microRNA action on vascular endothelial

**Figure 3:**
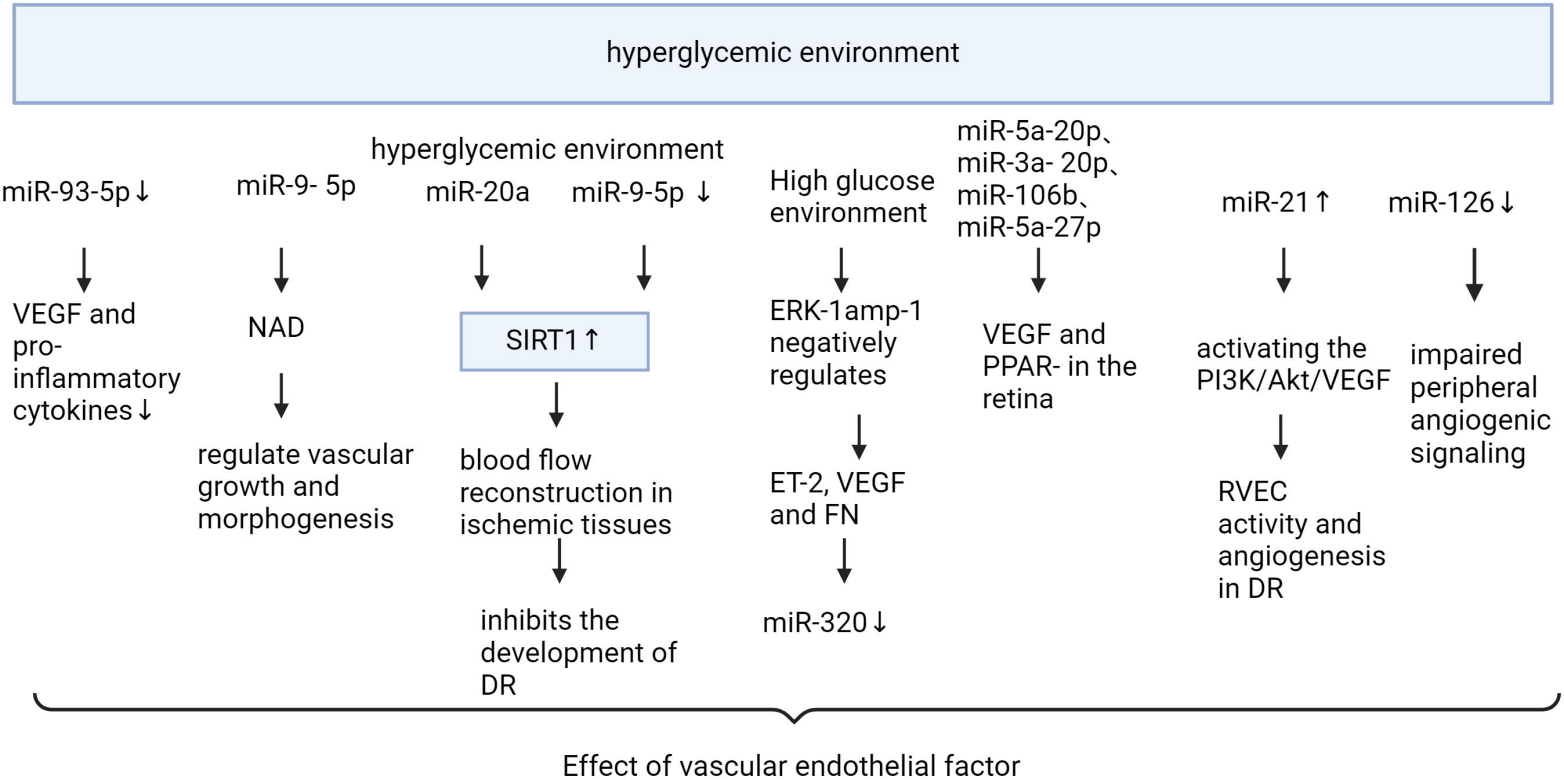
Mechanism of miRNA action in diabetic retinopathy through vascular endothelial factor “Created with BioRender.com”.

microRNAs affect diabetic retinopathy by modulating vascular endothelial factors (**Figure 3**, **Table 3**). VEGF is an essential angiogenic growth factor that increases vascular permeability and promotes the proliferation of vascular endothelial cells. Overexpression of miR-351 significantly reduces VEGF and Angiotensin II expression levels (73). The levels of VEGF and pro-inflammatory cytokines were reduced by miR-93-5p inhibition or Sirt1 overexpression in the retina of rats in the T2DM group, while the activity of antioxidant indicators was enhanced (74). Specific miR-9- 5p is associated with nicotinamide adenine dinucleotide (NAD) metabolism and senescence pathways that regulate vascular growth and morphogenesis (75). miR-20a upregulates SIRT1, which inhibits blood flow re-establishment in ischemic tissues and the development of DR (76). miR-148a-3p increases cell viability and reduces apoptosis by targeting Transforming growth factor β2 (TGFB2) and fibroblast β2 (FGF2), avoids damage to the blood-retinal barrier, and inhibits angiogenesis (77). Overexpression of miR-21 can stimulate retinal vascular endothelial cells (RVEC) and promote angiogenesis in DR rats by activating the PI3K/Akt/VEGF signaling pathway. MiR-21 also interferes with the expression of superoxide dismutase 2, thereby affecting the antioxidant response system (78). Metformin can downregulate the expression levels of anti-apoptotic miR-21 and miR-155 (89). After 3 months of treatment with empagliflozin, miR-21 and miR-92 were significantly reduced and endothelial dysfunction was alleviated in patients with heart failure with preserved ejection fraction (90). MiR-18b (79), miR-20b-5p (80), and miR-425-5p (91) promoted HRMEC proliferation and migration. Down-regulated MiR-126-3p in diabetic rat retina attenuates experimental diabetic retinopathy by targeting PLK4 to inhibit endothelial cell proliferation and migration (81). microRNA-126-5p promotes endothelial proliferation by inhibiting Dlk1, ultimately preventing atherosclerosis (82). miR-126 was reduced in DM (92), and delivery of functional miR-126-3p to recipient cells promoted vascular endothelial repair (83). Niaspan, an extended-release formulation of niacin, is safe for use in the treatment of diabetic patients, where it increases retinal miR-126 expression, inhibits inflammation, and promotes vascular remodeling (93). Trimetazidine improves endothelial dysfunction, increases plasma MicroRNA-24 and MicroRNA-126 levels, and ameliorates dyslipidemia, inflammation, and hypotension in diabetic rats (94). Upregulation of miR-126-5p attenuates apoptosis in human umbilical vein endothelial cells (HUVECs) by downregulating the PI3K/AKT/mTOR signaling pathway, alleviating endothelial cells injury in atherosclerosis (84). miR-126-5p activates the PI3K-AKT pathway by targeting IL-17A, reduces caspase-3 expression, promotes proliferation and inhibits apoptosis in HRECs (85). miR-126-5p regulates angiogenesis by inhibiting SetD5, which negatively controls class 3 semaphorin protein (Sema3A) in the RGC, and reduces endothelial cells of the retinal vascular system from apoptosis (86). The study demonstrated that a 5-day swimming regimen for 10 consecutive weeks significantly increased miR-126 expression, inhibited Sprouty-related EVH1 domain containing 1 and Phosphoinositide-3-kinase regulatory subunit 2 proteins, and improved coronary blood flow and function in rats (95). miR-221 and miR-222 were found to inhibit endothelial cell migration, proliferation, and angiogenesis *in vitro* by targeting stem cell factor receptor c-kit and indirectly regulating endothelial nitric oxide synthase expression. Downregulation of miR-222 by metformin exerts maintenance of endothelial integrity and cardioprotective effects. MiR-15a/16 maintains the retinal endothelial cell barrier by reducing TGFbeta3/VEGF signaling and increasing levels of key tight junction proteins (87). Low expression of microRNA-15b promotes the proliferation of retinal capillary endothelial cells and pericytes by upregulating VEGFA in diabetic rats (88).

**Table 3:** Mechanism of miRNA action in diabetic retinopathy through vascular endothelial factor.

Name	Dysregulation	Possible signaling pathways	Pathogenic functions	Reference
miR-351	upregulated		reduces VEGF and Ang-2 expression levels	(73)
miR-93-5p	downregulated		reduced VEGF and pro-inflammatory cytokines	(74)
miR-9- 5p	downregulated	NAD metabolism and senescence pathways	regulate vascular growth and morphogenesis	(75)
miR-20a	upregulated	SIRT1	inhibits blood flow re-establishment	(76)
miR-148a-3p	upregulated	TGFB2 and FGF2	Protects the blood-retinal barrier from damage and prevents the formation of new blood vessels	(77)
miR-21	upregulated	PI3K/Akt/VEGF	RVEC activity and angiogenesis	(78)
miR-18b	downregulated	insulin growth factor-1 receptor	promotes VEGF synthesis and HRECs proliferation	(79)
miR-20b-5p	upregulated	Bone morphogenetic protein and Activin Membrane-Bound Inhibitor	promoted proliferation, migration, and tube formation of HRMECs	(80)
MiR-126-3p	upregulated	PLK4	inhibit endothelial cell proliferation and migration	(81)
miR-126-5p	upregulated	Dlk1	Maintaining the proliferation reserve in EC	(82)
miR-126-3p	upregulated	SPRED1	vascular endothelial cell repair	(83)
miR-126-3p	upregulated	PI3K/AKT/mTOR	attenuates apoptosis in human aortic endothelial cells	(84)
miR-126-5p	upregulated	activates the PI3K-AKT pathway by targeting IL-17A, reduces caspase-3 expression	promotes proliferation and inhibits apoptosis in HRECs	(85)
miR-126-5p	upregulated	SetD5 and Sema3A	reduces endothelial cells of the retinal vascular system from apoptosis	(86)
miR-221 and miR-222	downregulated	stem cell factor receptor c-kit and indirectly regulating endothelial nitric oxide synthase expression	inhibit endothelial cell migration, proliferation, and angiogenesis *in vitro*	(87)
microRNA-15b		VEGFA	promotes the proliferation of retinal capillary endothelial cells	(88)

## 8 Summing up and looking ahead

MicroRNAs play an important regulatory role in diabetic retinal neurodegeneration and vascular degeneration. From the above account, it is clear that there is a close coupling between the retinal nervous system and the vascular cells, and that the metabolic needs of retinal neurons are preserved through the vascular system to maintain normal neural function. It is interesting to note that microRNAs have a dual effect on neurotrophic support by regulating vascular reconstruction and distribution, which in turn affects the nutritional support of the retinal nervous system.

A lot of research has been done to understand how microRNAs can cause retinal neuropathy. This can involve oxidative stress, apoptosis, inflammation, and disruptions in how the eye handles glutamate or a mix of these factors. In the high glucose environment, the expression level of microRNAs in the diabetic retina changed, and specific microRNAs appeared in the peripheral serum and vitreous. miRNAs in the form of exosomal vesicles or circulating miRNAs regulate relevant target cells and receptors in the retinal vasculature and nerves in patients with diabetes. Up- or down-regulated miRNAs affect the NF-κB pathway leading to decreased expression of antioxidant response-related molecules, increased iron death, and ROS generation. At the same time miRNAs lead to decreased vascular repair, proliferation of endothelial migration, and impaired vascular barrier by affecting vascular endothelial factor mechanisms. This leads to inflammation and injury in the retinal vasculature, ultimately leading to endothelial cell overgrowth and mobilization, vascular leakage, and neointimal formation. Increased levels of certain miRNAs, such as miRNA146a, miR-138- 5p, miR-338-3p and miR-126-5p can help reduce this damage. The new blood vessels are unable to provide proper nourishment for the surrounding tissues, while the leakage from the blood vessels disrupts the connection between the retinal nerves and blood vessels, leading to hypoxia in nerve cells. This hypoxia causes oxidative stress in photoreceptors, apoptosis of nerve cells, and the release of harmful substances by nerve cells. Moreover, miRNAs affected by high sugar levels can directly contribute to oxidative stress, affecting RPE and photoreceptors. At this stage, the patient experiences reduced sensitivity to light and impaired vision. A check-up may reveal a thinning of the retinal nerve fiber layer. The toxic substances released by nerve cells further contribute to disorders in the vascular system and inflammatory responses, creating a harmful cycle between nerve and vascular damage. Therefore, microRNAs may serve as mediators linking diabetic retinal neurodegeneration and vascular degeneration and become important targets for further research.

Most of the effects of microRNAs on the diabetic retinal vasculopathy are based on animal studies, and there’s a lack of research on their direct clinical applications. This article explains the alleviation of diabetic retinopathy and vascular disease by microRNAs by influencing apoptosis, oxidative stress, NF-kB inflammatory pathways, and endothelial factors. However, the article has not yet included all the microRNAs and downstream molecular mechanisms associated with diabetic retinoneuroangiopathy. microRNA-Mediated insulin signaling plays a key role in the pathological process of diabetic retinopathy, but the relevant miRNA is not discussed in this paper. An important area of future research in improving vision and quality of life for diabetic patients is the study of how miRNA functions in diabetic retinopathy and the development of new treatment approaches. Exosomes, which have membrane permeability, low immunogenicity, superior biodistribution, and biocompatibility, show potential as carriers for delivering RNA or targeted drugs due to their stability. There is particular interest in using exosomes to deliver specific miRNAs and investigating treatments to restore neurological functions. There are serious challenges in drug clinical trial studies of miRNA, and there is a need to improve miRNA target selectivity, specificity, and sensitivity, and to develop improved drug delivery methods. In addition, studies have shown that certain active ingredients in traditional Chinese medicine have the potential to reverse the aberrant regulation of miRNAs by the hyperglycemic environment, and future research in this area could be increased in order to provide a new avenue for the treatment of diabetic retinopathy.

## Author contributions

HZ: Writing – original draft, Writing – review & editing. YC: Investigation, Writing – review & editing. JP: Investigation, Writing – review & editing. QC: Methodology, Writing – review & editing.
